# A proof-of-concept study on the use of a fluorescein-based ^18^F-tracer for pretargeted PET

**DOI:** 10.1186/s41181-022-00155-2

**Published:** 2022-03-03

**Authors:** Hugo Helbert, Emily M. Ploeg, Douwe F. Samplonius, Simon N. Blok, Ines F. Antunes, Verena I. Böhmer, Gert Luurtsema, Rudi A. J. O. Dierckx, Ben L. Feringa, Philip H. Elsinga, Wiktor Szymanski, Wijnand Helfrich

**Affiliations:** 1 Stratingh Institute for Chemistry, University of Groningen, Nijenborgh 4, 9747 Groningen, The Netherlands; 2 Department of Nuclear Medicine and Molecular Imaging, Medical Imaging Center, University of Groningen, UMC Groningen, Hanzeplein 1, 9713 GZ Groningen, The Netherlands; 3 Department of Radiology, Medical Imaging Center, University of Groningen, UMC Groningen, Hanzeplein 1, 9713 GZ Groningen, The Netherlands; 4 Department of Surgery, Translational Surgical Oncology, University of Groningen, UMC Groningen, Hanzeplein 1, 9713 GZ Groningen, The Netherlands

**Keywords:** ImmunoPET, Pretargeting, [^18^F]Fluorescein, Bispecific antibody, [^18^F]TPF

## Abstract

**Background:**

Pretargeted immuno-PET tumor imaging has emerged as a valuable diagnostic strategy that combines the high specificity of antibody-antigen interaction with the high signal and image resolution offered by short-lived PET isotopes, while reducing the irradiation dose caused by traditional ^89^Zr-labelled antibodies. In this work, we demonstrate proof of concept of a novel ‘two-step’ immuno-PET pretargeting approach, based on bispecific antibodies (bsAbs) engineered to feature dual high-affinity binding activity for a fluorescein-based ^18^F-PET tracer and tumor markers.

**Results:**

A copper(I)-catalysed click reaction-based radiolabeling protocol was developed for the synthesis of fluorescein-derived molecule **[**^**18**^**F]TPF**. Binding of **[**^**18**^**F]TPF** on FITC-bearing bsAbs was confirmed. An in vitro autoradiography assay demonstrated that **[**^**18**^**F]TPF** could be used for selective imaging of EpCAM-expressing OVCAR3 cells, when pretargeted with EpCAMxFITC bsAb. The versatility of the pretargeting approach was showcased in vitro using a series of fluorescein-binding bsAbs directed at various established cancer-associated targets, including the pan-carcinoma cell surface marker EpCAM, EGFR, melanoma marker MCSP (aka CSPG4), and immune checkpoint PD-L1, offering a range of potential future applications for this pretargeting platform.

**Conclusion:**

A versatile pretargeting platform for PET imaging, which combines bispecific antibodies and a fluorescein-based ^18^F-tracer, is presented. It is shown to selectively target EpCAM-expressing cells in vitro and its further evaluation with different bispecific antibodies demonstrates the versatility of the approach.

**Supplementary Information:**

The online version contains supplementary material available at 10.1186/s41181-022-00155-2.

## Introduction

Driven by the established broad clinical applicability of ^18^F-FDG scans (Sheikhbahaei et al. 2017), positron emission tomography (PET) has become a leading technique for imaging of aberrant metabolic processes in the human body (Unterrainer et al. 2020). Diagnostic PET imaging aims to visualize disease-specific processes and/or markers using an appropriate selective radiotracer (Kilbourn et al. 2021). Typically, a PET radiotracer is a small target-seeking molecule equipped with a short-lived β^+^-emitting radioisotope. Amongst suitable isotopes for PET imaging, ^18^F is routinely used due of its convenient half-life (109.8 min) and low positron energy (E_mean_ = 0.250 MeV), which ensures high image resolution (Conti and Eriksson 2016). Considering the half-life of radionuclides, PET-tracers carrying them are required to possess compatible pharmacokinetics properties, *i.e.* fast biodistribution and accumulation in the tissue of interest, to enable optimal selective imaging after minutes up to few hours post-injection. Therefore, large target-seeking molecules, such as antibodies, constitute far from ideal candidates for the construction of PET tracers. Typically, antibodies, while offering selective, efficient, rapid, and sustained target binding, have impractically long circulation times of up to days, which limits their use in immuno-PET tumor imaging, particularly with respect to difficult-to-penetrate solid tumours. While the long half-life of ^89^Zr (78.4 h) has been successfully used for direct labelling of antibodies (Heskamp et al. 2017; McKnight and Viola-Vilegas et al. 2018), this strategy suffers from major impediments related to the long circulating time of the ^89^Zr-PET probe, notably the high irradiation dose received by healthy tissues (Jauw et al. 2016; Dewulf et al. 1868).

To overcome the limitations of using conventional antibodies for immuno-PET imaging, promising pretargeting approaches have been developed (Altai et al. 2017; Bailly et al. 2017; van de Watering et al. 2014; Marquez and Lapi 2016). Typically, pretargeting involves the injection into the circulation of the patient with an unlabelled, tumour-seeking, bi-functional antibody, which is given time (typically 24–48 h) to reach its optimal target binding at acceptable off-target background levels. Subsequently, a low molecular weight tracer is injected that is labelled with a positron-emitting radioligand for PET imaging, equipped with an inert binding tag (hapten) that can be efficiently captured by bifunctional antibodies that have accumulated at the site(s) of the tumour(s), whereas any unbound tracer rapidly clears from the circulation. Importantly from the application point of view, it is conceivable to administer the bispecific antibody outside of the specialized imaging department, after which the ^18^F-labeled agent is administered at the hospital site, shortly before the imaging procedure.

Pretargeting strategies notably exploit the strong biotin-streptavidin interaction (Weber et al. 1989; Hnatowich et al. 1987; Rusckowski et al. 1997; Goldenberg et al. 2006) and, in more recent examples, take advantage of the biorthogonal reaction of tetrazines with a *trans*-cyclooctenes (Rossin et al. 2010; Rossin and Robillard 2014; Rondon and Degoul 2020). Alternatively, pretargeting can effectively be achieved in vivo using so-called bispecific antibodies (bsAbs) (Molema et al. 2000; Lütje et al. 2014; Yu et al. 2017). BsAbs represent a unique class of rapidly emerging therapeutics that combine two target functionalities into one recombinant antibody-based molecule. In this respect, bsAbs have been engineered in which the primary binding domains are directed towards a preselected cancer-associated cell membrane marker and the secondary binding domains towards an inert small molecule (e.g. hapten). Upon selective binding and accumulation of the bsAb at the tumour site(s), the secondary binding domains are available for capturing a haptenylated compound of choice. Previously, pretargeting approaches have been described in which the tumour-directed bsAbs showed second specificity towards a chelator incorporated with a radiometal (Le Doussal et al. 1990), or a histamine-succinyl-glycine (HSG)-labelled tracer (Janevik-Ivanovska et al. 1997; Sharkey et al. 2012; Schoffelen et al. 2013).

## Results and discussion

### Design and synthesis

**Fig. 1:**
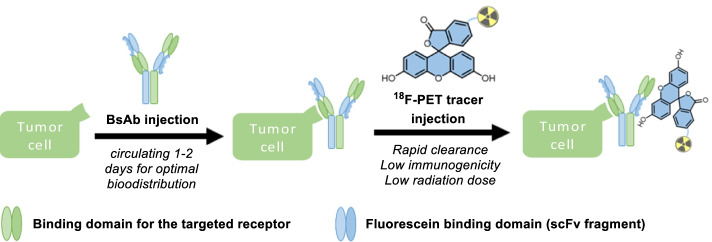
Immuno-PET pretargeting approach using fluorescein-based tracers. A bispecific antibody (containing two different scFv antibody fragments) is administered and allowed to circulate for 1–2 days for optimal biodistribution. Then, a labelled fluorescein-based ^18^F PET-tracer is administered, followed by PET data acquisition

**Scheme 1:**
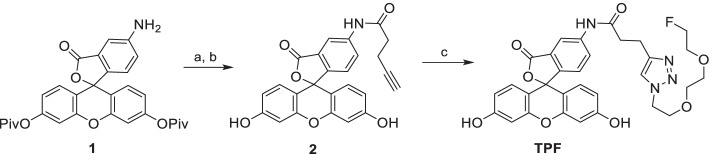
Synthesis of the TPF reference compound, employing CuAAC as the key labelling step. **a** 4-pentynoic acid, oxalyl chloride, DMAP, NEt_3_, DCM, rt, overnight (62%). **b** NaOH in MeOH, rt, 2 h (82%). **c** 1-azido-2-(2-(2-fluoroethoxy)ethoxy)ethane (**3**), CuSO_4_.5H_2_O, sodium ascorbate, TBTA, DMSO, 75 °C, 1 h (64%)

Here, we present a proof-of-concept for the use of a novel pretargeting platform, for PET imaging, combining bispecific antibodies (bsAbs) and a fluorescein-based ^18^F-PET tracer (Fig. 1). We selected fluorescein as a particularly suitable hapten for this approach, since it is an established, non-toxic, FDA-approved agent that is routinely used in angiography and that has confirmed low immunogenicity (Lumbroso et al. 2014). To construct a versatile platform for fluorescein-based pretargeted immuno-PET, we equipped bsAb with affinity-maturated scFv antibody fragments (K_D_ = 1.8 × 10^–10^ M) that potently bind to fluorescein and several derivatives thereof, including carboxy-fluorescein, fluorescein isothiocyanate (FITC), and Oregon green, and retain the binding potency even when these are conjugated to other compounds (Schwesinger et al. 2000; He et al. 2017). For the design of the fluorescein-based, ^18^F-bearing tracer, an alkyne moiety was introduced to the fluorescein molecule, enabling rapid labelling using copper(I) azide-alkyne cycloaddition (CuAAC) reaction (Meyer et al. 2016; Gill et al. 2011), to link the modified fluorescein with a fluorinated polyethylene glycol (PEG) chain as shown in Scheme 1 (Sirion et al. 2007; Li et al. 2007). The choice of ^18^F for the PET-tracer is noteworthy as the majority of previously mentioned strategies utilize higher energy radiometals combined with bispecific antibodies (Le Doussal et al. 1990; Janevik-Ivanovska et al. 1997; Sharkey et al. 2012; Schoffelen et al. 2013). The reference compound, triazole-pegylated-fluorescein (**TPF**), was prepared first (for details of the synthesis and characterization, see Additional file 1).

### In vitro binding assessment of TPF with bsAb EpCAMxFITC

Preliminary binding studies were performed between the non-radiolabeled **TPF** and the bsAb EpCAMxFITC that was engineered to have dual high-affinity binding activity for the pan-carcinoma cell surface marker EpCAM and fluorescein. Upon capture by bsAb EpCAMxFITC, the fluorescence of **TPF** is quenched, which enables the monitoring of the binding in real-time (Saunders et al. 2014). Titration of a **TPF** solution with bsAb EpCAMxFITC resulted in a significant reduction of the fluorescence signal (see Additional file 1: Figure S2). This effect was also observed when the bsAb EpCAMxFITC was first bound to EpCAM-expressing OVCAR3 cancer cells (see Additional file 1: Figure S3), indicating that **TPF** binds to bsAb EpCAMxFITC even at lower concentrations of bsAbs, relevant in pretargeting approaches, in this case limited by EpCAM expression. The selective binding of bsAb EpCAMxFITC to the cell surface of EpCAM-expressing OVCAR3 cancer cells was confirmed by flow cytometry (see Additional file 1: Figure S1).

### ***Radiolabelling of [***^***18***^***F]TPF***

**Scheme 2:**
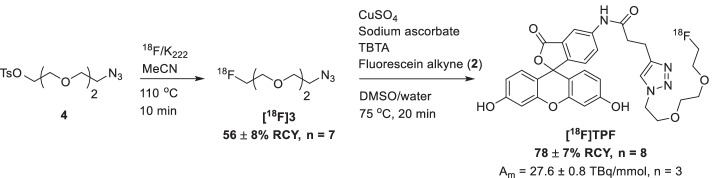
Radiosynthesis of [^18^F]TPF. The fluorescein-based tracer for PET pretargeting is obtained by radiolabeling of precursor **4** towards prosthetic group **[**^**18**^**F]3** and its subsequent CuAAC reaction with alkyne **2**

Next, the radiosynthesis of **[**^**18**^**F]TPF** was developed to allow for a rapid and efficient labelling procedure (Scheme 2). [^18^F]F^−^ (3–5 GBq) was azeotropically dried, before the addition of the tosylate precursor **4** (3 mg, 9 µmol) in MeCN. The ^18^F-fluorination was carried out for 10 min at 110 ºC. The labelled azide **[**^**18**^**F]3** was isolated by HPLC in 56 ± 8% radiochemical yield (RCY, n = 7, see Additional file 1: Figure S4 for radio-TLC analysis and Additional file 1: Figure S4 for the HPLC analysis), concentrated in an HLB Plus Short cartridge, eluted with 1.2 mL of DMSO, and further reacted with the fluorescein alkyne **2** (1.0–1.5 mg, 2.3–3.5 µmol**)** in the presence of a premixed catalytic mixture containing CuSO_4_, sodium ascorbate and TBTA. The CuAAC reaction allowed for the synthesis of **[**^**18**^**F]TPF** in high yields (78 ± 7% RCY, n = 8) with a molar activity of A_m_ = 27.6 ± 0.8 TBq/mmol (n = 3), and high radiochemical purity (> 99%). The detailed labelling procedure is available in Additional file 1, together with the HPLC analysis of the reaction (Additional file 1: Figure S6) and product purity (Additional file 1: Figure S7). The in vitro stability of **[**^**18**^**F]TPF** was evaluated indicating no measurable degradation after incubation in human plasma at 37 °C for 2 h (see Additional file 1: Figure S8).

In this study we focused exclusively on employing **[**^**18**^**F]TPF** in combination with FITC-containing bsAb for pretargeting purposes. Nonetheless, capitalizing on the diverse uses of fluorescein derivatives, **[**^**18**^**F]TPF** can prove to be relevant beyond this application. For example, taking advantage of the fluorescent properties of **[**^**18**^**F]TPF** offers opportunities in dual-modality imaging, combining a PET signal with optical imaging (Jennings et al. 2009). Alternative applications for **[**^**18**^**F]TPF** also includes the sensitive detection of cerebrospinal fluid leaks by PET imaging (Kommidi et al. 2017; Guo et al. 2019), where similar fluorescein-based ^18^F-PET tracers have emerged in the recent years.

### In vitro*** pretargeting assay with [***^***18***^***F]TPF on EpCAM-expressing OVCAR3 cells***

**Fig. 2:**
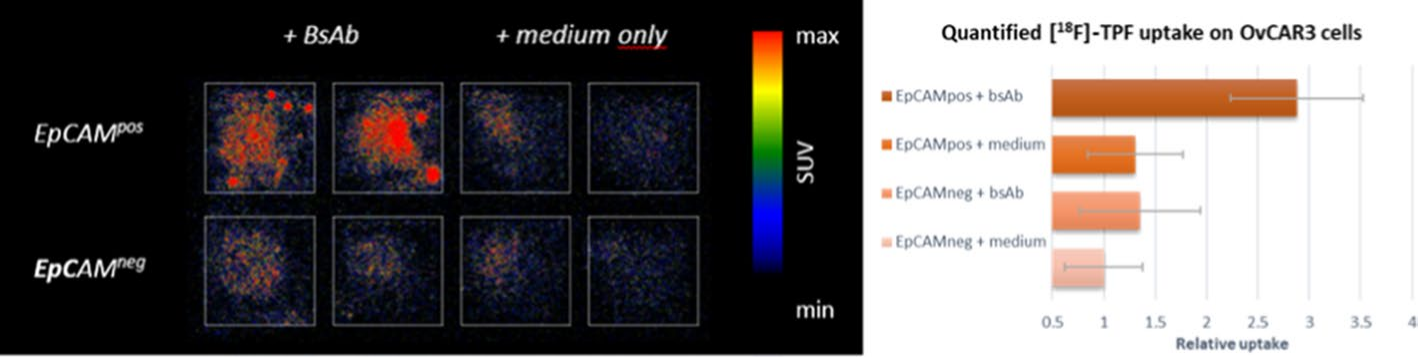
Autoradiography assessment of the capacity of cancer cell-bound bsAb EpCAMxFITC to capture [^18^F]TPF. See Additional file 1: Section 11 for experimental details

The radiotracer **[**^**18**^**F]TPF** was used in a radio-assay to confirm binding to bsAb EpCAMxFITC. Immobilizing of bsAb EpCAMxFITC on protein A agarose beads and comparing the uptake of **[**^**18**^**F]TPF** with a similarly constructed bsAb EpCAMxMock enabled the evaluation of binding specificity. A nine-fold increase in binding was observed for the bsAb EpCAMxFITC compared with EpCAM-binding bsAb with an irrelevant second binding specificity (bsAb EpCAMxMock) (Additional file 1: Figure S9). Next, a pretargeting experiment was executed using EpCAM-expressing OVCAR3 cancer cells pretargeted (or not) with bsAb EpCAMxFITC, washed to remove any unbound antibody, and then incubated with **[**^**18**^**F]TPF**. After a final washing step to remove any unbound **[**^**18**^**F]TPF**, the radiotracer uptake was evaluated. To demonstrate EpCAM-selectivity of this approach, control experiments were performed using OVCAR3 cancer cells in which EpCAM expression was knocked out using CRISPR-Cas9 gene editing technology. Visualization and quantification of bsAb-mediated uptake of **[**^**18**^**F]TPF** was performed by autoradiography of the cancer cells (Fig. 2). The low, and similar, **[**^**18**^**F]TPF** uptake observed for both EpCAM^neg^ and EpCAM^pos^ cells that where not incubated with bsAb, confirms that **[**^**18**^**F]TPF** does not bind directly to EpCAM and is not subject to unspecific uptake by cells, both being important criteria for a suitable PET-tracer. The selectivity of the EpCAMxFITC bsAb for the EpCAM and the efficiency of the washing procedure is demonstrated by the low **[**^**18**^**F]TPF** uptake observed for EpCAM^neg^ cells incubated with bsAb that did not show a significant difference compared with the uptake observed for EpCAM^neg^ cells not incubated with this bsAb. Finally, the results of the autoradiography indicated enhanced uptake of **[**^**18**^**F]TPF** by EpCAM-positive OVCAR3 cancer cells when these cells were pretargeted with bsAb EpCAMxFITC, compared to all three control groups (n = 12, P < 0.001). This result indicates that **[**^**18**^**F]TPF** retains its high affinity for the EpCAMxFITC bsAb, even when the bsAb is already engaged in binding with EpCAM, an essential indication that this approach is suitable for pretargeting of EpCAM-expressing cancer cells. These results suggest that EpCAM-selective PET tumour imaging may be achieved in a two-step pretargeting approach using bsAb EpCAMxFITC and **[**^**18**^**F]TPF**.

### ***[***^***18***^***F]TPF uptake by a variety of bsAbs***

**Fig. 3:**
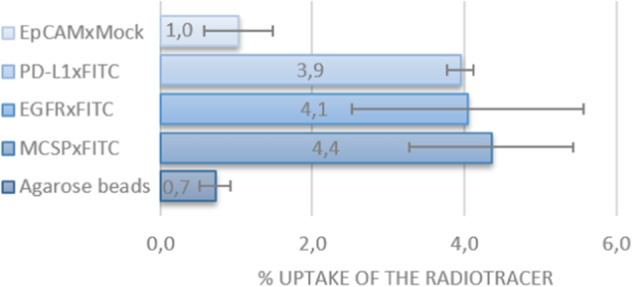
Capture of [^18^F]TPF by bsAbs immobilized on Protein A agarose beads. See Additional file 1: Section 10 for experimental details

To demonstrate the versatility of our bsAb-based pretargeted immuno-PET platform, we extended our study with additional FITC-binding bsAbs, each directed at clinically relevant cancer-associated target molecules, including melanoma marker MCSP (aka CSPG4), epidermal growth factor receptor (EGFR) and immune checkpoint PD-L1. In particular, bsAb MCSPxFITC was engineered to have high affinity binding capacity for the  cell surface molecule MCSP, a promising target for malignant melanoma (Kageshita et al. 1991; Jordaan et al. 2017). Analogously, bsAb EGFRxFITC was engineered to have high affinity binding capacity for EGFR, a clinically relevant cancer-associated cell surface marker that is selectively overexpressed on various difficult-to-treat carcinomas and glioma (Sun et al. 2018). Finally, we evaluated the applicability of our pretargeting strategy using bsAb PD-L1xFITC that was engineered to have high-affinity blocking capacity for the inhibitory immune checkpoint molecule PD-L1, which is frequently overexpressed in various cancer types. Imaging of PD-L1-expressing tumors may be of therapeutic value to identify cancer patients who may profit from cancer immunotherapy based on PD-L1 immune checkpoint blockade (Koopmans et al. 2019; van de Donk et al. 2020; Koopmans et al. 2018). Gratifyingly, target antigen-selective binding was observed for all the studied bsAbs. Specifically, incubation of each of these bead-immobilized bsAbs with the **[**^**18**^**F]TPF** radiotracer resulted in an up to four-fold increase in binding of **[**^**18**^**F]TPF** by the corresponding bsAb compared with control bsAb EpCAMxMock, which was not equipped with the anti-fluorescein scFv fragment responsible for **[**^**18**^**F]TPF** binding (Fig. 3). Noticing no significant difference in **[**^**18**^**F]TPF** uptake between the bsAb EpCAMxMock and the protein A agarose beads only, we can confidently exclude binding of the **[**^**18**^**F]TPF** independent of the anti-fluorescein scFv fragment. Importantly, there was no significant difference in **[**^**18**^**F]TPF** uptake for all bsAbs containing this fluorescein-binding scFv fragment, suggesting that modifying the bsAb format to direct it towards different cell surface tumor markers does not influence **[**^**18**^**F]TPF** binding by the anti-fluorescein scFv fragment. These results indicate the versatility of our bsAb-based two-step PET imaging approach for various tumour types using one and the same **[**^**18**^**F]TPF** tracer.

## Conclusion

In conclusion, the study of a novel pretargeted immuno-PET platform based on a combination of bispecific antibodies and a ^18^F-fluorescein-based PET-probe showed promising results in vitro. An efficient radiolabeling protocol, exploiting copper-catalyzed click chemistry, was developed for the radiosynthesis of **[**^**18**^**F]TPF**. The ^18^F-modified-Fluorescein tracer retained its affinity for FITC bsAbs and the specific uptake of **[**^**18**^**F]TPF** in EpCAM-overexpressing OVCAR3 cells, pretargeted with EpCAMxFITC bsAb, was confirmed by in vitro autoradiographic assays. These results should encourage further development and optimization of **[**^**18**^**F]TPF** for in vivo ovarian cancer imaging applications. Lastly, the versatility of the pretargeting approach was demonstrated by **[**^**18**^**F]TPF** capture by a series of cancer-selective bsAbs, targeting clinically relevant cancer-associated cell surface markers MSCP, PD-L1 and EGFR. We envision that this novel and versatile bsAb-based approach may prove to be useful to broaden the clinical efficacy of immuno-PET tumour imaging.

## Methods

### General and synthetic methods

For the general description of the materials, cell lines, methods and synthetic procedures, please see Sections 1–3 of the Additional file 1.

### Antibody production and binding assessment

For the general description of the antibody construction, eucaryotic production and the assessment of binding and florescence quenching capacity, see Sections 4–7 of the Additional file 1.

### Radiolabeling of [^18^F]TPF

A QMA cartridge was pre-conditioned by passing 10 mL of 1.4% aq. sol. NaHCO_3_ followed by 15 mL of deionized H_2_O to reach pH = 7. The cartridge was then dried under argon flow. The [^18^F]F^−^ in enriched [^18^O]H_2_O obtained from the cyclotron (typically 3–5 GBq in 1.2 mL) was passed through the dried cartridge, followed by 5 mL of air to elute most of the H_2_O. The [^18^F]F^−^ remaining on the QMA was eluted, into a 4 mL conical vial, with a 1 mL solution containing 15 mg of Kryptofix K_222_ in 800 μL of MeCN and 1 mg of K_2_CO_3_ in 200 μL H_2_O. Solvents were removed at 115 °C under magnetic stirring and argon flow, 1 mL of anhydrous MeCN was added and evaporated to dryness. This process was repeated 3 times with 0.5 mL of anhydrous MeCN. To the azeotropically dried [^18^F]F^−^/K_222_ mixture was added 3 mg (9 μmol) of 2-(2-(2-azidoethoxy)ethoxy)ethyl 4-methylbenzenesulfonate (**5**) in 0.5 mL of anhydrous MeCN. (NB.: The tosylate **5** was previously azeotropically dried at 100 °C by repeated addition of anhydrous MeCN and evaporation (3 × 0.5 mL)). The reaction mixture was then stirred at 110 °C for 10 min and, at the end of the reaction, was diluted with 0.5 mL of H_2_O. The reaction mixture was then injected on HPLC (see Additional file 1: Section 8 for HPLC conditions) and the product (**[**^**18**^**F]3**) was collected (t_R_ = 8.2 min., 56 ± 8% RCY from dry [^18^F]F^−^). The collected fraction was diluted with 80 mL of H_2_O and concentrated in an Oasis PRiME HLB Plus Short cartridge, which was pre-conditioned with 10 mL of H_2_O. In the meantime, a catalytic mixture was prepared for the CuAAC reaction. First, 4 mg (20 μmol) of sodium ascorbate in 200 μL of H_2_O were added to 2.5 mg (10 μmol) Cu_2_SO_4_^.^5H_2_O dissolved in 100 μL of H_2_O. The resulting solution was pre-mixed for 5 min to obtain a dark brown solution. 5.6 mg (10.5 μmol) of TBTA in 200 μL of DMSO was added and mixed to afford a light orange solution, from which 200 μL was taken and added to a 4 mL conical vial containing 1.5 mL of fluorescein alkyne (**4**). **[**^**18**^**F]3** was eluted from the HLB cartridge into the same 4 mL conical vial by passing 1.2 mL of DMSO through the HLB cartridge. The reaction mixture was stirred at 80 °C for 20 min. At the end of the reaction, 0.8 mL of H_2_O (containing 0.1% of formic acid) was added and the solution was purified by HPLC (see Additional file 1: Section 8 for HPLC conditions) and the **[**^**18**^**F]TPF** product was collected (t_R_ = 23 min, 78 ± 7% RCY from eluted **[**^**18**^**F]3**). The collected fraction was diluted with 70 mL of H_2_O and concentrated in a pre-conditioned (10 mL of EtOH followed by 20 mL of H_2_O) Waters Sep-Pak Plus Light C18 cartridge. **[**^**18**^**F]TPF** was eluted from cartridge with 1 mL of EtOH and diluted with 9 mL of PBS affording typically 300–700 MBq of product. A sample was taken and submitted to UPLC measurement for QC to assess the purity and molar activity of the product. All the radio-TLC and HPLC analyses can be found in the Additional file 1: Section 8.

### Stability [^18^F]TPF in human serum

In an Eppendorf tube, containing 200 μL of human serum, was added 5 μL of **[**^**18**^**F]TPF** (50 kBq) in PBS/EtOH (9/1). The mixture was shaken at 37 °C for 0, 60 and 120 min. 200 μL of MeCN was the added to induce protein precipitation. The sample was then vortexed and the supernatant was analyzed on an iTLC and compared with the initial **[**^**18**^**F]TPF** (Rf = 0.45; 10% MeOH in DCM as eluent). After exposure to a phosphor plate, the iTLC was read on an Amersham Typhoon Imager (see Additional file 1: Figure S8).

### Assessment of capacity of bead-bound bsAb EpCAMxFITC to capture [^18^F]TPF

In short, 10 μg of bsAb EpCAMxFITC (or bsAb EpCAMxMock) was mixed with a slurry of 50 µl Protein A agarose beads (SinoBiological) in final volume of 500 µl PBS, and the mixture incubated at rt for 30 min. Next, the beads were pelleted by centrifugation (2500 rpm, 5 min) and washed 2 times with PBS to remove unbound bsAb. The washed beads were resuspended in 100 μL PBS, mixed with 25 μL of a **[**^**18**^**F]TPF** (0.5–1.0 MBq) solution in PBS/EtOH (9:1 vol/vol), and incubated under continuous gentle shaking at rt for 15 min. Then, PBS was added to a final volume of 500 µl, after which the beads were pelleted by centrifugation (2500 rpm, 5 min). Next, 480 μL of the supernatant solution was collected and replaced by the same volume of PBS after which the beads were pelleted again. This washing procedure was repeated 2 more times after which radioactivity associated with beads and that present in the combined supernatants were then measured using a gamma counter.

### Assessment of capacity of cancer cell-bound bsAb EpCAMxFITC to capture [^18^F]TPF

In short, 10 × 10^3^ OVCAR-3 cells per well were seeded in an 8 wells permanox chamber slide (LAB-TEK, Nagle Nunc Int.), allowed to adhere, and then incubated (or not) with 10 μg of bsAb EpCAMxFITC (or bsAb EpCAMxMock) at 37 °C for 1 h. Next, the cells were washed 2 times with PBS to remove unbound bsAb and then incubated (or not) with 200 μL of 0.5–1 MBq of **[**^**18**^**F]TPF** in PBS (< 2% EtOH) at rt for 30 min. Subsequently, the cells were washed two times with PBS to remove unbound **[**^**18**^**F]TPF**. Finally, the growth chamber was carefully removed, after which the glass slide with adhered cells was carefully washed with PBS and then air-dried. The dried glass slide was wrapped in aluminium foil and exposed to a phosphor plate for up to 1 h. The phosphor plate (see Section 11 of the Additional file 1) was imaged using a Amersham Typhoon Imager to record the autoradiographic images.

## Supplementary Information

**Additional file 1**. General methods, Synthetic procedures, Radiolabeling procedures, NMR Spectra of the products, Data on the cell lines, Antibody construction and production. Additional file 1: Figure S1. Binding capacity of bsAb EpCAMxFITC for both EpCAM (A) and fluorescein (B). Additional file 1: Figure S2: bsAb EpCAMxFITC shows dose-dependent capacity to quench the fluorescein-mediated fluorescent activity of TPF, Additional file 1: Figure S3: Capacity of free bsAb EpCAMxFITC and cancer cell-bound bsAb EpCAMxFITC to capture TPF and quench its fluorescence. Additional file 1: Figure S4: Radio-TLC after the fluorination reaction to obtain [^18^F]3 (eluent: EtOAc/Hexane, 1/1). Additional file 1: Figure S5: HPLC Chromatogram of the purification of [^18^F]3. UV detector λ = 254 nm. Additional file 1: Figure S6: HPLC Chromatogram of the purification of [^18^F]TPF. UV detector λ = 312 nm. Additional file 1: Figure S7: HPLC Chromatogram of the collected peak for [^18^F]TPF, co-injected with the non-radiolabelled reference compound TPF. Additional file 1: Figure S8: Stability of [^18^F]TPF in human serum. Additional file 1: Figure S9: Assessment of the capacity of bead-bound bsAb EpCAMxFITC to capture [18F]TPF. Additional file 1: Figure S10: 8 wells permanox chamber slide with seeded cells. Additional file 1: Figure S11: ^1^H and ^13^C NMR spectra of compound 1. Additional file 1: Figure S12: ^1^H and ^13^C NMR spectra of compound S2. Additional file 1: Figure S13: ^1^H and ^13^C NMR spectra of compound 2. Additional file 1: Figure S11: ^1^H, ^13^C and ^19^F NMR spectra of TPF.

## Data Availability

The dataset(s) supporting the conclusions of this article is (are) included within the article (and its additional file(s)).

## Abbreviations

bsAb: Bispecific antibody
CSPG4: Chondroitin sulfate proteoglycan 4
CuAAC: Copper-Catalyzed Azide-Alkyne Cycloaddition
EGFR: Epidermal growth factor receptor
EpCAM: Epithelial cell adhesion molecule
FDG: Fluorodeoxyglucose
FITC: Fluorescein isothiocyanate
HPLC: High-performance liquid chromatography
MSCP: Melanoma chondroitin sulfate proteoglycan
PD-L1: Programmed death-ligand 1
PET: Positron Emission Tomography
RCY: Radiochemical yield

## References

[CR1] Altai M, Membreno R, Cook B, Tolmachev V, Zeglis BM (2017). Pretargeted imaging and therapy. J Nucl Med.

[CR2] Bailly C, Bodet-Milin C, Rousseau C, Faivre-Chauvet A, Kraeber-Bodéré F, Barbet J (2017). Pretargeting for imaging and therapy in oncological nuclear medicine. EJNMMI Radiopharm Chem.

[CR3] Conti M, Eriksson L (2016). Physics of pure and non-pure positron emitters for PET: a review and a discussion. EJNMMI Phys.

[CR4] Dewulf J, Adhikari K, Vangestel C, Van Den Wyngaert T, Elvas F (1868). Development of antibody immuno-PET/SPECT radiopharmaceuticals for imaging of oncological disorders—an update. Cancers.

[CR5] Gill H, Marik J (2011). Preparation of ^18^F-labeled peptides using the copper(I)-catalyzed azide-alkyne 1,3-dipolar cycloaddition. Nat Protoc.

[CR6] Goldenberg DM, Sharkey RM, Paganelli G, Barbet J, Chatal JF (2006). Antibody pretargeting advances cancer radioimmunodetection and radioimmunotherapy. J Clin Oncol.

[CR7] Guo H, Kommidi H, Maachani UB, Voronina JC, Zhang W, Magge RS, Ivanidze J, Wu AP, Souweidane MM, Aras O, Ting R (2019). An [^18^F]-positron emitting fluorophore allows safe evaluation of small molecule distribution in the CSF, CSF fistulas, and CNS device placement. Mol Pharm.

[CR8] He Y, van Bommel PE, Samplonius DF, Bremer E, Helfrich W (2017). A versatile pretargeting approach for tumour-selective delivery and activation of TNF superfamily members. Sci Rep.

[CR9] Heskamp S, Raavé R, Boerman O, Rijpkema M, Goncalves V, Denat F (2017). ^89^Zr-immuno-positron emission tomography in oncology: state-of-the-art ^89^Zr radiochemistry. Bioconjugate Chem.

[CR10] Hnatowich DJ, Virzi F, Rusckowski M (1987). Investigations of avidin and biotin for imaging applications. J Nucl Med.

[CR11] Janevik-Ivanovska E, Gautherot E, Hillairet de Boisferon M, Cohen M, Mil-haud G, Tartar A, Rostene W, Barbet J, Gruaz-Guyon A (1997). Bivalent hapten-bearing peptides designed for iodine-131 pretargeted radioimmunotherapy. Bioconj Chem.

[CR12] Jauw YWS, Menke-van der Houven van Oordt CW, Hoekstra OS, Hendrikse NH, Vugts DJ, Zijlstra JM, Huisman MC, van Dongen GAMS (2016). Immuno-positron emission tomography with zirconium-89-labeled monoclonal antibodies in oncology: what can we learn from initial clinical trials?. Front Pharmacol.

[CR13] Jennings LE, Long NJ (2009). ‘Two is better than one’—probes for dual-modality molecular imaging. Chem Commun.

[CR14] Jordaan S, Chetty S, Mungra N, Koopmans I, van Bommel PE, Helfrich W, Barth S (2017). CSPG4: a target for selective delivery of human cytolytic fusion proteins and TRAIL. Biomedicines.

[CR15] Kageshita T, Nakamura T, Yamada M, Kuriya N, Arao T, Ferrone S (1991). Differential expression of melanoma associated antigens in acral lentiginous melanoma and in nodular melanoma lesions. Cancer Res.

[CR16] Kilbourn MR, Scott PJH (2021). Handbook of radiopharmaceuticals: methodology and applications.

[CR17] Kommidi H, Guo H, Chen N, Kim D, He B, Wu AP, Aras O, Ting R (2017). An [^18^F]-positron-emitting, fluorescent, cerebrospinal fluid probe for imaging damage to the brain and spine. Theranostics.

[CR18] Koopmans I, Hendriks D, Samplonius DF, van Ginkel RJ, Heskamp S, Wierstra PJ, Bremer E, Helfrich W (2018). A novel bispecific antibody for EGFR-directed blockade of the PD-1/PD-L1 immune checkpoint. Oncoimmunology.

[CR19] Koopmans I, Hendriks MAJM, van Ginkel RJ, Samplonius DF, Bremer E, Helfrich W (2019). Bispecific antibody approach for improved melanoma-selective PD-L1 immune checkpoint blockade. J Investig Dermatol.

[CR20] Le Doussal JM, Gruaz-Guyon A, Martin M, Gautherot E, De Laage M, Barbet J (1990). Targeting of indium 111-labeled bivalent Hapten to human melanoma mediated by bispecific monoclonal antibody conjugates: imaging of tumors hosted in nude mice. Cancer Res.

[CR21] Li ZB, Wu Z, Chen K, Chin FT, Chen X (2007). Click chemistry for (18)F-labeling of RGD peptides and microPET imaging of tumor integrin alphavbeta3 expression. Bioconjug Chem.

[CR22] Lumbroso B, Rispoli M (2014). Practical handbook of fluorescein angiography.

[CR23] Lütje S, Rijpkema M, McBride B, Sharkey R, Goldenberg D, Franssen G, Eek A, Helfrich W, Oyen W, Boerman O (2014). Pretargeted dual-modality SPECT/fluorescence imaging of prostate cancer with an anti-TROP-2 x anti-HSG bispecific antibody. J Nucl Med.

[CR24] Marquez BV, Lapi SE (2016). Pretargeted immuno-PET: overcoming limitations of space and time. J Nucl Med.

[CR25] McKnight BN, Viola-Villegas NT (2018). ^89^Zr-ImmunoPET companion diagnostics and their impact in clinical drug development. J Label Compd Radiopharm.

[CR26] Meyer JP, Adumeau P, Lewis JS, Zeglis BM (2016). Click chemistry and radiochemistry: the first 10 years. Bioconjugate Chem.

[CR27] Molema G, Jan Kroesen B, Helfrich W, Meijer DKF, de Leij LFMH (2000). The use of bispecific antibodies in tumor cell and tumor vasculature directed immunotherapy. J Control Release.

[CR28] Rondon A, Degoul F (2020). Antibody pretargeting based on bioorthogonal click chemistry for cancer imaging and targeted radionuclide therapy. Bioconjugate Chem.

[CR29] Rossin R, Robillard MS (2014). Pretargeted imaging using bioorthogonal chemistry in mice. Curr Opin Chem Biol.

[CR30] Rossin R, Renart Verkerk P, van den Bosch SM, Vulders RCM, Verel I, Lub J, Robillard MS (2010). In vivo chemistry for pretargeted tumor imaging in live mice. Angew Chem Int Ed.

[CR31] Rusckowski M, Fogarasi M, Fritz B, Hnatowich DJ (1997). Effect of endogenous biotin on the applications of streptavidin and biotin in mice. Nucl Med Biol.

[CR32] Saunders MJ, Block E, Sorkin A, Waggoner AS, Bruchez MP (2014). A bifunctional converter: fluorescein quenching scFv/fluorogen activating protein for photostability and improved signal to noise in fluorescence experiments. Bioconjugate Chem.

[CR33] Schoffelen R, Boerman OC, Goldenberg DM, Sharkey RM, van Herpen CML, Franssen GM, McBride WJ, Chang CH, Rossi EA, van der Graaf WTA, Oyen WJG (2013). Development of an imaging-guided CEA-pretargeted radionuclide treatment of advanced colorectal cancer: first clinical results. Br J Cancer.

[CR34] Schwesinger F, Ros R, Strunz T, Anselmetti D, Güntherodt HJ, Honegger A, Jermutus L, Tiefenauer L, Plückthun A (2000). Unbinding forces of single antibody-antigen complexes correlate with their thermal dissociation rates. PNAS.

[CR35] Sharkey RM, van Rij CM, Karacay H, Rossi EA, Frielink C, Regino C, Cardillo TM, McBride WJ, Chang CH, Boerman OC, Goldenberg DM (2012). A new tri-fab bispecific antibody for pretargeting trop-2—expressing epithelial cancers. J Nucl Med.

[CR36] Sheikhbahaei S, Marcus CV, Fragomeni RS, Rowe SP, Javadi MS, Solnes LB (2017). Whole-body 18 F-FDG PET and 18 F-FDG PET/CT in patients with suspected paraneoplastic syndrome: a systematic review and meta-analysis of diagnostic accuracy. J Nucl Med.

[CR37] Sirion U, Kim HJ, Lee JH, Seo JW, Lee BS, Lee SJ, Oh SJ, Chi DY (2007). An efficient F-18 labeling method for PET study: Huisgen 1,3-dipolar cycloaddition of bioactive substances and F-18-labeled compounds. Tetrahedron Lett.

[CR38] Sun X, Xiao Z, Chen G, Han Z, Liu Y, Zhang C, Sun Y, Song Y, Wang K, Fang F, Wang X, Lin Y, Xu L, Shao L, Li J, Cheng Z, Gambhir SS, Shen B (2018). A PET imaging approach for determining EGFR mutation status for improved lung cancer patient management. Sci Transl Med.

[CR39] Unterrainer M, Eze C, Ilhan H, Marschner S, Roengvoraphoj O, Schmidt-Hegemann NS, Walter F, Kunz WG, Munck af Rosenschöld P, Jeraj R, Albert NL, Grosu AL, Niyazi M, Bartenstein P, Belka C (2020). Recent advances of PET imaging in clinical radiation oncology. Radiat Oncol.

[CR40] van de Donk PP, Kist de Ruijter L, Lub-deHooge MN, Brouwers AH, van der Wekken AJ, Oosting SF, Fehrmann RSN, de Groot DJA, de Vries EGE (2020). Molecular imaging biomarkers for immune checkpoint inhibitor therapy. Theranostics.

[CR41] van de Watering FC, Rijpkema M, Robillard M, Oyen WJ, Boerman OC (2014). Pretargeted imaging and radioimmunotherapy of cancer using antibodies and bioorthogonal chemistry. Front Med.

[CR42] Weber PC, Ohlendorf DH, Wendoloski JJ, Salemme FR (1989). Structural origins of high-affinity biotin binding to streptavidin. Science.

[CR43] Yu S, Li A, Liu Q, Yuan X, Xu H, Jiao D, Pestell RG, Han X, Wu K (2017). Recent advances of bispecific antibodies in solid tumors. J Hematol Oncol.

