# Supplementary material for: A proof-of-concept study on the use of a fluorescein-based 18F-tracer for pretargeted PET
Source: EJNMMI Radiopharm Chem. 2022 Mar 3;7:3. doi: 10.1186/s41181-022-00155-2 (PMC8894538; doi:10.1186/s41181-022-00155-2)

Electronic Supporting Information

A versatile pretargeting PET imaging platform combining bispecific antibodies with a fluorescein-based ^{18}F-PET tracer

Hugo Helbert[a,b], Emily M. Ploeg[d], Douwe F. Samplonius[d], Simon N. Blok[b], Ines Farinha Antunes[a], Verena I. Böhmer[a,b], Gert Luurtsema[b], Rudi A.J.O. Dierckx[b], Ben L. Feringa[a], Philip H. Elsinga[b], Wiktor Szymanski[c] and Wijnand Helfrich[d]

[a]*Stratingh Institute for Chemistry, University of Groningen, The Netherlands*
[b]*Department of Nuclear Medicine and Molecular Imaging, University of Groningen, University Medical Center Groningen, The Netherlands*
[c]*Department of Radiology University of Groningen, University Medical Center Groningen, The Netherlands*
[d]*Department of Surgery, Translational Surgical Oncology, University Medical Center Groningen, The Netherlands*

Contents

1. General methods 2
2. Experimental procedures for the synthesis of TPF 3
3. Cell lines 5
4. Construction of bsAb EpCAMxFITC 6
5. Eukaryotic production of bsAb EpCAMxFITC 6
6. Assessment dual binding capacity of bsAb EpCAMxFITC for EpCAM and fluorescein 6
7. Assessment fluorescence quenching capacity of bsAb EpCAMxFITC 8
8. Radiolabelling of [^{18}F]TPF 9
9. Stability [^{18}F]TPF in human serum 12
10. Assessment of capacity of bead-bound bsAb EpCAMxFITC to capture [^{18}F]TPF 12
11. Assessment of capacity of cancer cell-bound bsAb EpCAMxFITC to capture [^{18}F]TPF . 13
12. NMR-spectra 14

1. General methods

Column chromatography: Grace-Reveleris purification system with Büchi silica cartridges. TLC: Merck silica gel (60, 0.25 mm). Compounds were detected on TLC using UV light (254 nm, 365 nm) and visualized by potassium permanganate staining. Mass spectra were recorded on a LTQ Orbitrap XL (ESI+). ^{1}H- and ^{13}C-NMR were recorded on a Bruker 600 (at 600 and 151 MHz), a Varian AMX400 (at 400 and 100.59 MHz) and at 300 MHz (300, 75 MHz) for ^{1}H- and ^{13}C-NMR, using appropriate solvents ($CDCl_3$, DMSO-*d6*, MeOD-*d4*. Chemical shift values are reported in ppm with the solvent resonance as the internal standard. Data are reported as follows: chemical shifts, multiplicity (s = singlet, d = doublet, t = triplet, q = quartet, m = multiplet), coupling constants (Hz), and integration. Fluorine-18 (^{18}F) was produced by the nuclear reaction ^{18}O(p,n)^{18}F using an IBA Cyclone 18/18 cyclotron. High-performance liquid chromatography (HPLC) chromatograms were acquired on a Waters system using a 1525 binary HPLC pump, a 2489 UV/visible detector, and a Berthold Technologies Flowstar LB 513 radio flow detector. Ultra-high performance liquid chromatography (UPLC) spectra were acquired using a Waters Acquity UPLC integrated system coupled to a Berthold Technologies Flowstar LB 513 radio flow detector. HPLC and UPLC data were processed with Waters Empower 3 software. Radio-TLC's and the cells-on-glass-slides used for autoradiography were scanned using a GE Amersham Typhoon 5 Biomolecular Imager by GE Healthcare Life Sciences, and the acquired data ware analysed with OptiQuant 3.0 software or ImageQuant TL software. Radioactivity of samples was counted for 15 s using a WIZARD® Automatic Gamma Counter 2470, PerkinElmer. Data were analysed for statistical significance by t-test student test using SigmaPlot 14 software.

2. Experimental procedures for the synthesis of TPF

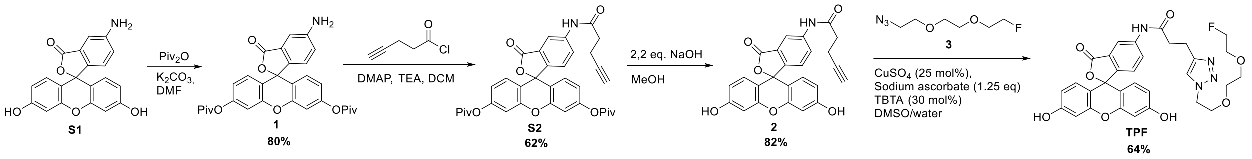

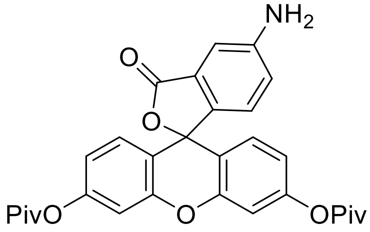

1: 5-Amino-3-oxo-3H-spiro[isobenzofuran-1,9'-xanthene]-3',6'-diyl bis(2,2-dimethylpropanoate). Prepared by a modification of a literature procedure.[1] A mixture of 5-aminofluorescein (710 mg, 2.05 mmol, 1.0 eq), pivalic anhydride (1.33 mL, 6.55 mmol, 3.2 eq) and potassium carbonate (0.86 g, 6.34 mmol, 3.1 eq) in DMF (12 mL) was stirred at rt for 3 h. The reaction mixture was diluted with ethyl acetate (60 mL) and washed with brine (4 x 50 mL). The organic phase was dried over $MgSO_4$ and filtered. The volatiles were evaporated and the product was purified by column chromatography (pentane/Et_2O, gradient from 1/1 to pure Et_2O) to give a white powder (850 mg, 80 %). R_f = 0.77 (Et_2O); Mp. 140-142 °C; ^{1}H NMR (400 MHz, $CDCl_3$): δ 1.35 (s, 18H, Piv**H**), 4.07 (s, 2H, N**H**$_2$), 6.76 (dd, ^3J = 8.8 Hz, ^4J = 2.4 Hz, 2H, Ar**H**), 6.87 (d, ^3J = 8.8 Hz, 2H, Ar**H**), 6.90 (d, ^3J = 8.4 Hz, 1H, Ar**H**), 6.94 (dd, ^3J = 8.4 Hz, ^4J = 2.0 Hz, 1H, Ar**H**), 7.02 (d, ^4J = 2.4 Hz, 2H, Ar**H**), 7.19 (d, ^4J = 2.0 Hz, 1H, Ar**H**); ^{13}C NMR (100 MHz, $CDCl_3$): δ 27.1, 39.2, 81.7, 108.6, 110.1, 116.9, 117.5, 122.4, 124.7, 127.7, 129.0, 142.8, 148.3, 151.7, 152.4, 169.5, 176.5; HRMS (ESI+) calc. for $[M+H]^+$ ($C_{30}H_{30}NO_7$): 516.2017, found: 516.2013.

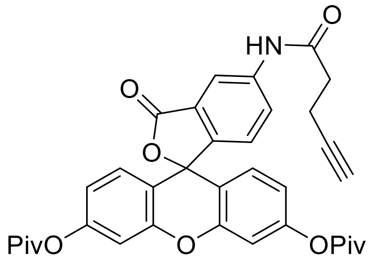

S2: 3-Oxo-5-(pent-4-ynamido)-3H-spiro[isobenzofuran-1,9'-xanthene]-3',6'-diyl bis(2,2-dimethylpropanoate). A solution of pentynoic acid (0.90 mmol, 88 mg), oxalyl chloride (2 mmol,

[1] Hishikawa, K.; Nakagawa, H.; Furuta, T.; Fukuhara, K.; Tsumoto, H.; Suzuki, T.; Miyata, N. *J. Am. Chem. Soc.* **2009**, *131*, 7488–7489

172 µL) and DMF (1 drop) in DCM (5 mL) was stirred at rt for 1 h. The volatiles were evaporated, and the residue was dissolved in DCM (2 mL). This solution was added dropwise to a solution of compound **1** (0.50 mmol, 257 mg), triethylamine (1.00 mmol, 138 µL) and DMAP (10 mg) in DCM (8 mL). The resulting reaction mixture was stirred at rt overnight. The volatiles were evaporated, and the product was purified by column chromatography (pentane/AcOEt, 4/1) to give a white powder (187 mg, 62 %). Mp = 197-199 °C. R_f = 0.80 (pentane/AcOEt, 1/1); ^{1}H NMR (400 MHz, $CDCl_3$): δ 1.35 (s, 18H, Piv**H**), 2.02 (t, ^4J = 2.4 Hz, 1H, CC**H**), 2.55-2.65 (m, 4H, C**H$_2$**C**H$_2$**), 6.76 (dd, ^3J = 8.8 Hz, ^4J = 2.4 Hz, 2H, Ar**H**), 6.82 (d, ^3J = 8.4 Hz, 2H, Ar**H**), 7.04 (d, ^4J = 2.4 Hz, 2H, Ar**H**), 7.11 (d, ^3J = 8.4 Hz, 1H, Ar**H**), 8.02 (dd, ^3J = 8.4 Hz, ^4J = 2.0 Hz, 1H, Ar**H**), 8.19 (d, ^4J = 2.0 Hz, 1H, Ar**H**), 8.35 (s, 1H, N**H**); ^{13}C NMR (100 MHz, $CDCl_3$): δ 14.6, 27.0, 36.1, 39.2, 69.8, 82.2, 82.6, 110.3, 115.3, 116.1, 117.7, 124.6, 127.1, 127.1, 128.9, 140.1, 147.8, 151.7, 152.6, 169.1, 169.9, 176.6; HRMS (ESI+) calc. for $[M+H]^+$ ($C_{35}H_{34}NO_8$): 596.2279, found: 596.2278.

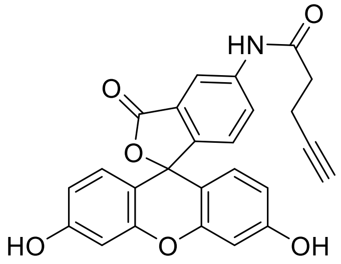

2: *N*-(3',6'-dihydroxy-3-oxo-3H-spiro[isobenzofuran-1,9'-xanthen]-5-yl)pent-4-ynamide. A solution of NaOH (21 mg, 0.53 mmol, 2.2 eq) in 4 mL of MeOH was added dropwise to compound **S2** (140 mg, 0.24 mmol, 1.0 eq) dissolved in 2 mL of MeOH, and the resulting solution was stirred at rt until TLC showed complete conversion (2 – 3 h). The reaction was quenched by addition of 2.0 M HCl sol. in Et_2O (0.30 mL, 0.60 mmol, 2.5 eq). The volatiles were evaporated, and the product was purified by column chromatography (DCM/MeOH, gradient from 97/3 to 90/10) to give a yellow powder (84 mg, 82 %). Mp = 160 °C (dec). ^{1}H NMR (400 MHz, DMSO-d_6): δ 2.45-2.53 (m, 2H, C**H$_2$**CCH) 2.57 (t, 2H, ^3J = 6.4 Hz, C**H$_2$**CO), 2.82 (t, ^4J = 2.4 Hz, 1H, CC**H**), 6.53 (dd, ^3J = 8.8 Hz, ^4J = 2.0 Hz, 2H, Ar**H**), 6.59 (d, ^3J = 8.4 Hz, 2H, Ar**H**), 6.66 (d, ^4J = 2.0 Hz, 2H, Ar**H**), 7.20 (d, ^3J = 8.4 Hz, 1H, Ar**H**), 7.81 (dd, ^3J = 8.4 Hz, ^4J = 2.4 Hz, 1H, Ar**H**), 8.20 (d, ^4J = 2.4 Hz, 1H, Ar**H**), 10.09 (s, 2H, O**H**), 10.44 (s, 1H, N**H**); ^{13}C NMR (100 MHz, DMSO-d_6): δ 14.5, 35.8, 72.0, 83.5, 83.9, 102.7, 110.2, 113.0, 113.8, 124.9, 126.7, 127.4, 129.5, 141.1, 147.2, 152.3, 159.9, 169.1, 170.5; HRMS (ESI+) calc. for $[M+H]^+$ ($C_{25}H_{18}NO_6$): 428.1129, found: 428.1128.

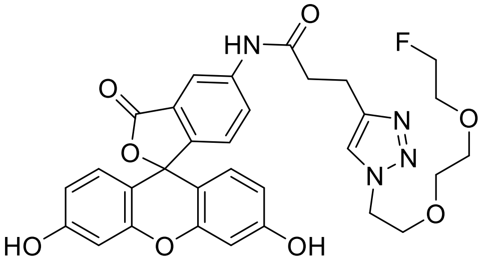

TPF: *N*-(3',6'-dihydroxy-3-oxo-3H-spiro[isobenzofuran-1,9'-xanthen]-5-yl)-3-(1-(2-(2-(2-fluoroethoxy)ethoxy)ethyl)-1H-1,2,3-triazol-4-yl)propanamide.

$Cu_2SO_4 \cdot 5H_2O$ (9.2 mg, 37 µmol, 0.25 eq) and sodium ascorbate (36 mg, 0.18 mmol, 1.25 eq) were pre-mixed in water (0.4 mL) for 10 min. Tris(benzyltriazolylmethyl)amine (TBTA, 9.2 mg, 0.44 mmol, 0.3 eq) was added in one portion and the solution was stirred for 5 min and then added to a solution of compound **2** (63 mg, 0.15 mmol, 1.00 eq) and 1-azido-2-(2-(2-fluoroethoxy)ethoxy)ethane (**3**) (41 mg, 0.18 mmol, 1.20 eq, prepared according to a published procedure[2]) in DMSO (1.5 mL). The reaction mixture was stirred at 75 °C for 1 h, after which it was diluted with AcOEt (50 mL) and washed with 1N HCl (4 x 30 mL). The organic phase was dried over $MgSO_4$ and filtered. The volatiles were evaporated, and the product was purified by column chromatography (DCM/MeOH, gradient from 95/5 to 80/20) to give a crude solid product. The solid was further triturated in pentane, the precipitate collected by filtration, and then washed with Et_2O to afford an orange solid powder (57 mg, 64 %). Mp ~120 °C (Dec.). ^{1}H NMR (400 MHz, CD_3OD): δ 2.86 (t, ^3J = 7.2 Hz, 2H, C**H$_2$**CO), 3.13 (t, ^3J = 7.2 Hz, 2H, C**H$_2$**CH$_2$CO), 3.58-3.62 (m, 4H, OC**H$_2$**C**H$_2$**O), 3.66 (dt, J_{HF} = 30.4 Hz, J_{HH} = 4.0, 2H, C**H$_2$**CH$_2$F), 3.88 (t, ^3J = 5.2 Hz, 2H, C**H$_2$**CH$_2$triazole), 4.49 (dt, J_{HF} = 48.4 Hz, J_{HH} = 4.0, 2H, C**H$_2$**F), 4.57 (t, ^3J = 5.2 Hz, 2H, C**H$_2$**triazole), 6.91 (d, ^3J = 8.0 Hz, 2H, Ar**H**), 7.06 (s, 2H, Ar**H**), 7.15 (d, ^3J = 8.8 Hz, 2H, Ar**H**), 7.28 (d, ^3J = 8.4 Hz, 1H, Ar**H**), 7.93 (s, 1H, triazole**H**), 7.99 (d, ^3J = 8.4 Hz, 1H, Ar**H**), 8.49 (s, 1H, Ar**H**); ^{13}C NMR (100 MHz, $CDCl_3$): δ20.4, 26.5, 35.6, 50.2, 68.9, 70.0, 70.0, 70.1, 70.2, 81.9, 83.5, 102.0, 114.0, 116.4, 118.7, 124.5, 127.8, 129.8, 131.1, 141.0, 156.5, 166.2, 167.8; ^{19}F NMR (376 MHz, $CDCl_3$): -224.5 (m); HRMS (ESI+) calc. for $[M+H]^+$ ($C_{31}H_{30}FN_4O_{10}$): 605.2042, found: 605.2037.

3. Cell lines

The EpCAM-expressing ovarian cancer cell line OVCAR-3 was obtained from the American Type Culture Collection (ATCC) and cultured in DMEM (Lonza), supplemented with 10% fetal calf serum (FCS, Thermo Scientific) at 37 °C in a humidified 5% CO_2 atmosphere.

OVCAR-3.EpCAM-KO cells were generated by transfection of parental OVCAR-3 cells with plasmid pSpCas9 BB-2A-GFP (PX458) containing the EpCAM CRISPR-cas9 targeting gRNA

[2] Rokka, J.; Snellman, A.; Zona, C.; La Ferla, B.; Nicotra, F.; Salmona, F.; Forloni, G.; Haaparanta-Solin, M.; Rinne, J. O.; Solin, O. *Bioorg. Med. Chem.* **2014**, *22*, 2753-2762.

sequence TAATGTTATCACTATTGATC after which OVCAR-3.EpCAM-KO cells were single cell sorted by flow cytometry.

4. Construction of bsAb EpCAMxFITC

DNA fragments encoding antibody fragments scFvEpCAM and scFvFITC were generated by commercial gene synthesis service (Genscript), based on the published VH and VL sequence data of the anti-EpCAM antibody 4D5 MOC-B[3] and the anti-fluorescein antibody 4-4-20,[4] respectively. For construction and production of bsAb EpCAMxFITC, we used a home-made eukaryotic expression plasmid pbsAb which contains 3 consecutive multiple cloning sites (MCS). MCS#1 and MCS#2 are interspersed by a 22 amino acid flexible linker derived from a CH1 IgG domain. MCS#1, MCS#2 and MCS#3 were used for directional and in-frame insertion of DNA fragments encoding scFvEpCAM, scFvFITC, and human IgG1 Fc domain, respectively, yielding plasmid pbsAb EpCAMxFITC (Suppl. Fig. X). Of note, this expression plasmid is equipped with a strong CMV promoter and a murine kappa light-chain leader peptide for high level expression of bsAb EpCAMxFITC through the ER and Golgi complex of Expi293 production cells, ensuring excretion of correctly folded and biological active recombinant bsAb with authentic port-translational modifications. Control bsAb EpCAMxMock was constructed and produced analogously replacing scFvFITC from bsAb EpCAMxFITC with a Mock scFv with irrelevant specificity directed to human CD20.

5. Eukaryotic production of bsAb EpCAMxFITC

BsAb EpCAMxFITC was produced using the Expi293 expression system (ThermoFisher). Briefly, Expi293 cells were transfected with plasmid pbsAb EpCAMxFITC and cultured on a shaker platform (125 rpm) at 37 °C, 8% CO_2, for 7 days. Culture supernatant was harvested and cleared by centrifugation (3000 x g, 30 min), after which bsAb EpCAMxFITC was purified using an HiTrap protein A HP column connected to an ÄKTA Start chromatography system (GE Healthcare Life Sciences).

6. Assessment dual binding capacity of bsAb EpCAMxFITC for EpCAM and fluorescein

EpCAM-selective binding capacity of bsAb EpCAMxFITC to cell surface-expressed EpCAM was confirmed by flow cytometry using parental OVCAR-3 vs. OVCAR-3.EpCAM-KO cells. Briefly, 10^5 OVCAR-3 or OVCAR-3.EpCAM-KO cells were incubated (or not) with bsAb EpCAMxFITC (5 µg/ml) at 4°C for 40 min. Next, the cells were washed and incubated with AF647-labelled goat-antihuman IgG at 4°C for 40 min. After washing the cells were analysed by flow cytometry (Merck, Guava Easycyte) (Figure S1.A).

[3] J Willuda, A Honegger, R Waibel, P A Schubiger, R Stahel, U Zangemeister-Wittke, A Plückthun *Cancer Res.* **1999**, *59* (22), 5758-5767.
[4] Jung S, Honegger A, Plückthun A. *J Mol Biol.* **1999**, *294* (1), 163-180.

Next, the ability of OVCAR-3 cell surface-bound bsAb EpCAMxFITC to capture FITC-labelled AnnexinV was assessed by flow cytometry. Briefly, 10^5 OVCAR-3 cells were incubated with AnnexinV-FITC (5 µg/ml) in the presence (or absence) of bsAb EpCAMxFITC (5 µg/ml) at 4°C for 40 min. After washing cells were analysed by flow cytometer. Of note, incubations with AnnexinV-FITC were performed in the absence of Ca^{2+} thereby reducing background binding of AnnexinV to phosphatidyl serine that may be exposed on the membrane of OVCAR-3 cells (Figure S1.B).

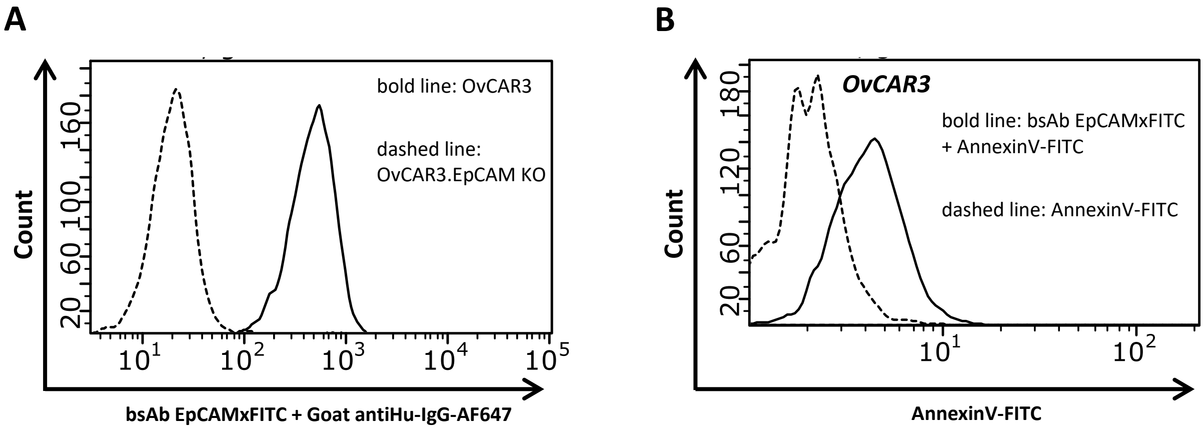

Supplementary Figure S1: Binding capacity of bsAb EpCAMxFITC for both EpCAM (A) and fluorescein (B)

7. **Assessment fluorescence quenching capacity of bsAb EpCAMxFITC**

The capacity of bsAb EpCAMxFITC to quench the fluorescence of the fluorescein domain of TPF in solution was tested as follows. In short, 50 µL TPF (0,2 µg/mL in PBS) was added per well of a 96 wells plate. Subsequently, increasing amounts of bsAb EpCAMxFITC (0 - 25 µg in PBS) were added to the individual wells and the final volume of each well was adjusted to 60 µL using PBS. Quenching of fluorescence in each well was assessed at λ_{ex} = 496 nm and λ_{em} = 518 nm using a microtiter plate reader (Perklin Elmer, Victor3).

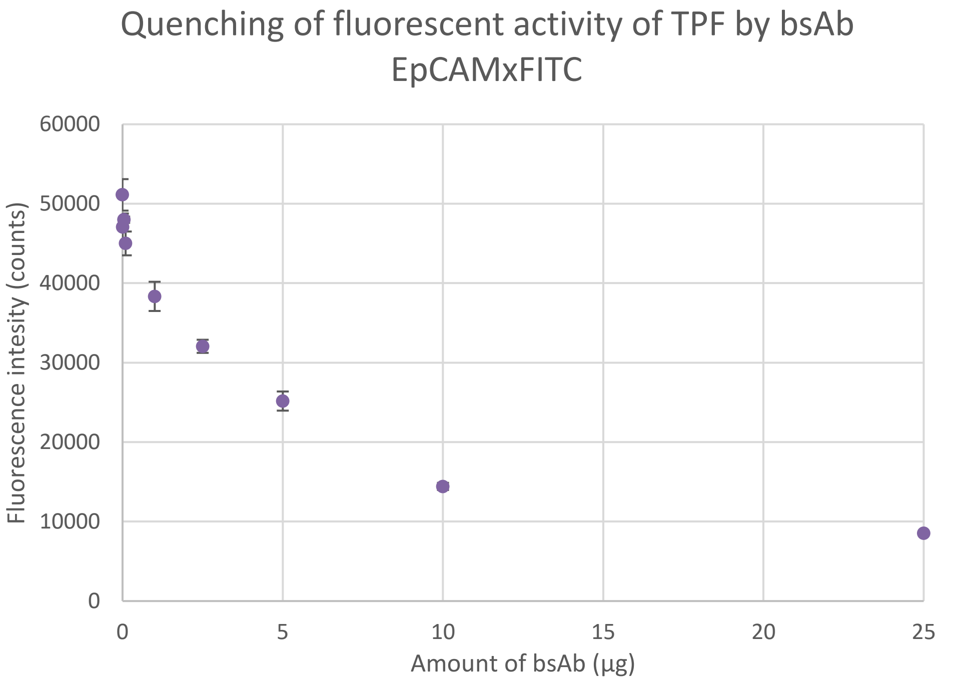

Supplementary Figure S2: bsAb EpCAMxFITC shows dose-dependent capacity to quench the fluorescein-mediated fluorescent activity of TPF.

The capacity of cancer cell-bound bsAb EpCAMxFITC to capture TPF and quench its fluorescent activity was tested as follows. In short, EpCAM-expressing OVCAR-3 cancer cells (~10^6 cells/eppendorf tube) were washed three times with PBS and then incubated (or not) with 10 µg bsAb EpCAMxFITC for 40 min, after which the unbound bsAb was removed by washing two times with PBS. Subsequently, cell pellets were resuspended in PBS in a final volume of 20 µL and then incubated (or not) with 100 µL TPF (0.2 µg/mL in PBS) at rt for 30 min. Next, cells were pelleted by centrifugation and 60 µl supernatant of each sample was transferred to a separate well of a 96 wells plate which was then scanned for remaining fluorescent activity.

Of note: Essentially the same experiment was performed in the absence of bsAb, the fluorescence intensity obtained from the experiment without bsAb was set as the 100% fluorescence and used to calculate the relative fluorescence intensity.

$$Relative\ fluorescence\ intensity = \frac{Fluorescence\ intensity\ with\ bsAb}{Fluorescence\ intensity\ without\ bsAb}$$

The data obtained were analysed by a t-test and the difference between experimental groups was considered significant when P = <0,001.

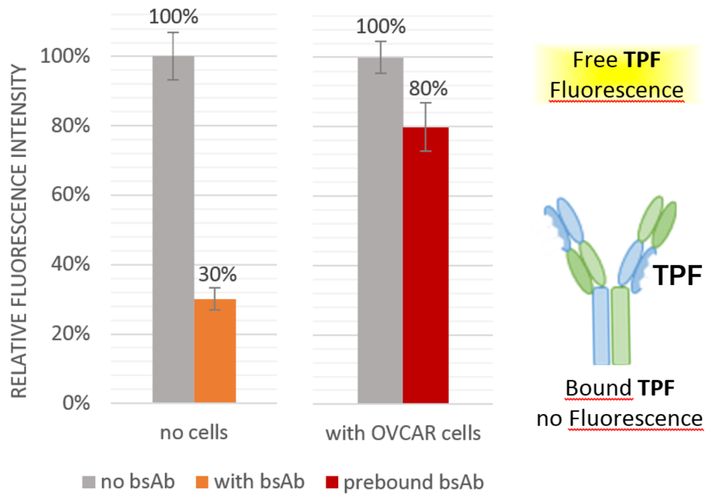

Supplementary Figure S3: Capacity of free bsAb EpCAMxFITC and cancer cell-bound bsAb EpCAMxFITC to capture TPF and quench its fluorescence.

8. Radiolabelling of [^{18}F]TPF

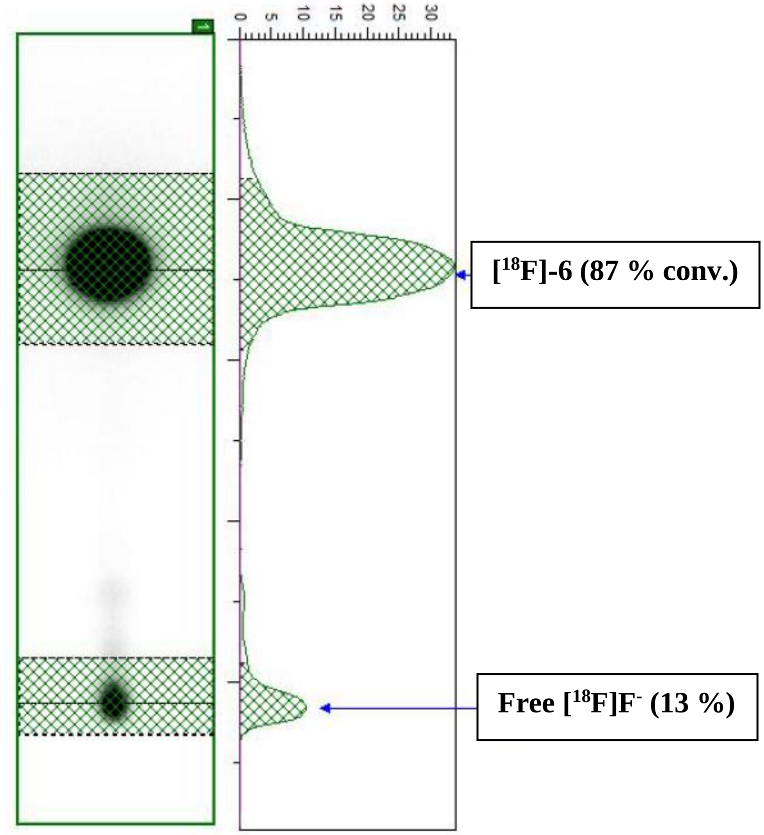

Supplementary Figure S4: Radio-TLC after the fluorination reaction to obtain **[^{18}F]3** (eluent: EtOAc/Hexane, 1/1)

<u>HPLC condition for **[^{18}F]3** purification :</u>

- Column: XBridge BEH Shield RP18, OBD Prep Column, 130Å, 5 µm, 10 mm x 250 mm
- Eluent: MeOH / H_2O (containing 0.1% of formic acid); isocratic : 30/70.
- Flow: 5 mL/min
- Retention time: 8.2 min

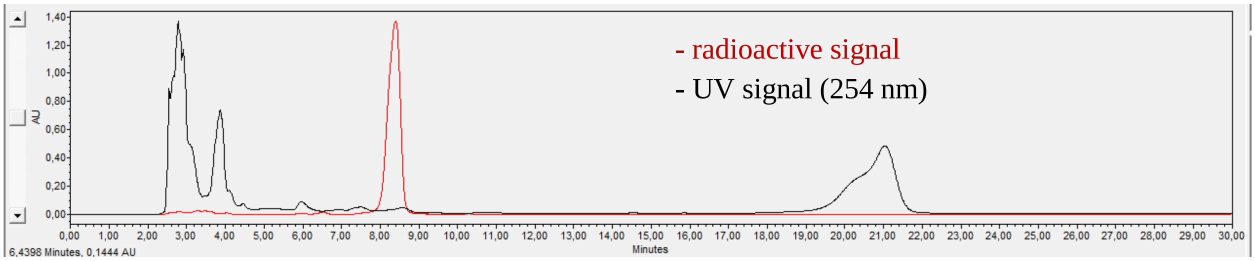

Supplementary Figure S5: HPLC Chromatogram of the purification of **[^{18}F]3**. UV detector λ = 254 nm

<u>HPLC condition for **[^{18}F]TPF** purification :</u>

- Column: XBridge BEH Shield RP18, OBD Prep Column, 130Å, 5 µm, 10 mm x 250 mm
- Eluent: MeOH / H_2O (containing 0.1% of formic acid);
- Gradient:

Time (min)	MeOH (vol %)	0.1% formic acid in H_2O (vol %)
0	40	60
4	40	60
15	60	40
35	90	10
40	90	10

- Flow: 3 mL/min
- Retention time: 23 min

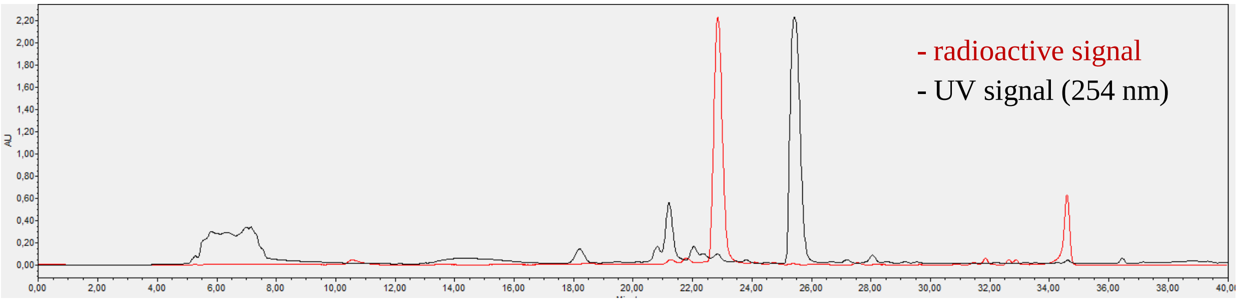

Supplementary Figure S6: HPLC Chromatogram of the purification of **[^{18}F]TPF.** UV detector λ = 312 nm

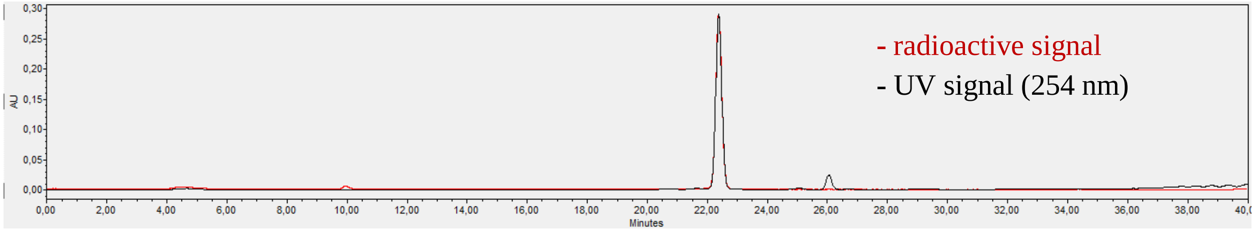

Supplementary Figure S7: HPLC Chromatogram of the collected peak for **[^{18}F]TPF**, co-injected with the non-radiolabelled reference compound **TPF**

9. Stability [^{18}F]TPF in human serum

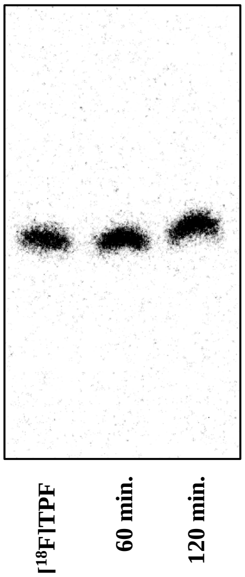

Supplementary Figure S8: Stability of [^{18}F]TPF in human serum

10. Assessment of capacity of bead-bound bsAb EpCAMxFITC to capture [^{18}F]TPF

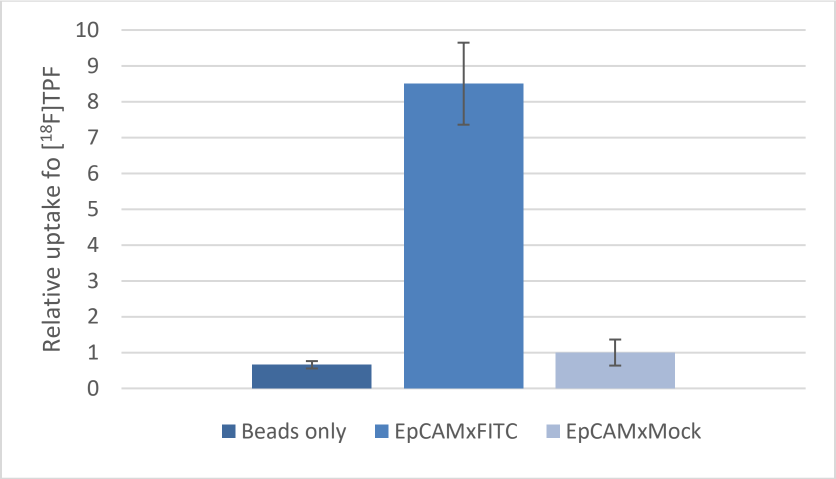

Supplementary Figure S9: Assessment of the capacity of bead-bound bsAb EpCAMxFITC to capture [^{18}F]TPF

11. Assessment of capacity of cancer cell-bound bsAb EpCAMxFITC to capture [^{18}F]TPF

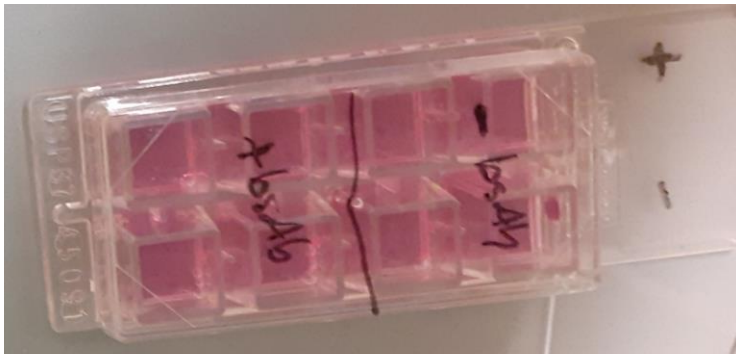

Supplementary Figure S10: 8 wells permanox chamber slide with seeded cells.

12. NMR-spectra

Supplementary Figure S11: ^{1}H and ^{13}C NMR spectra of compound **1**

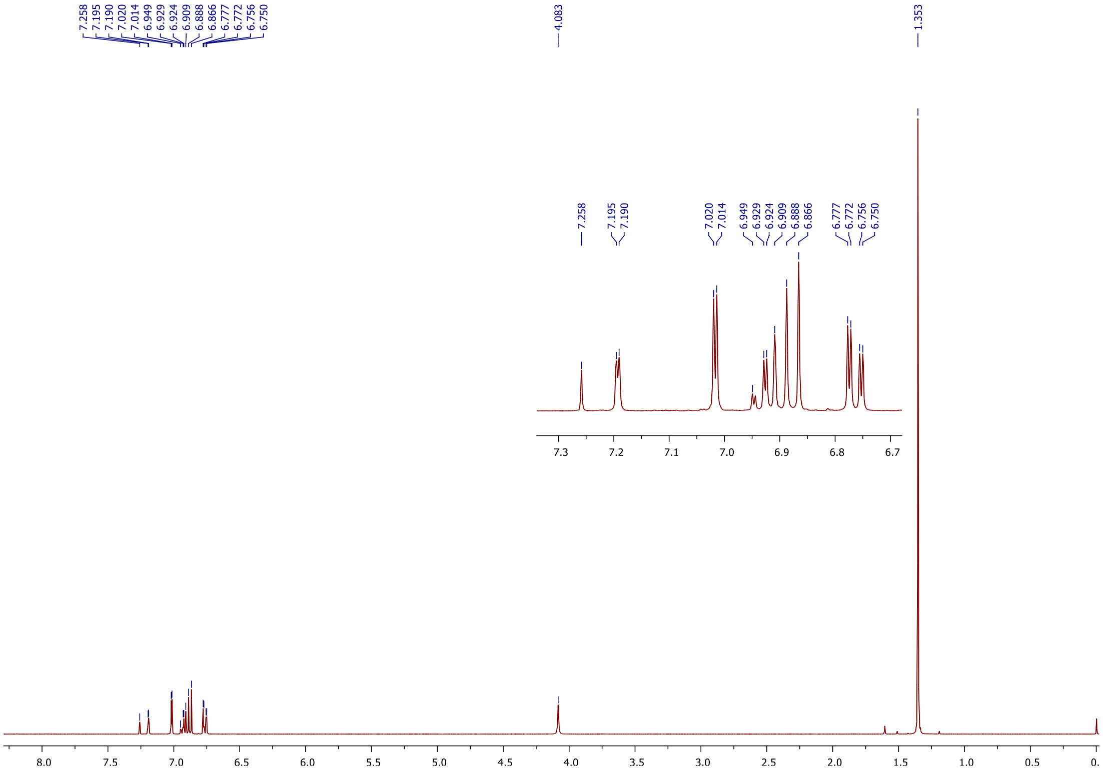

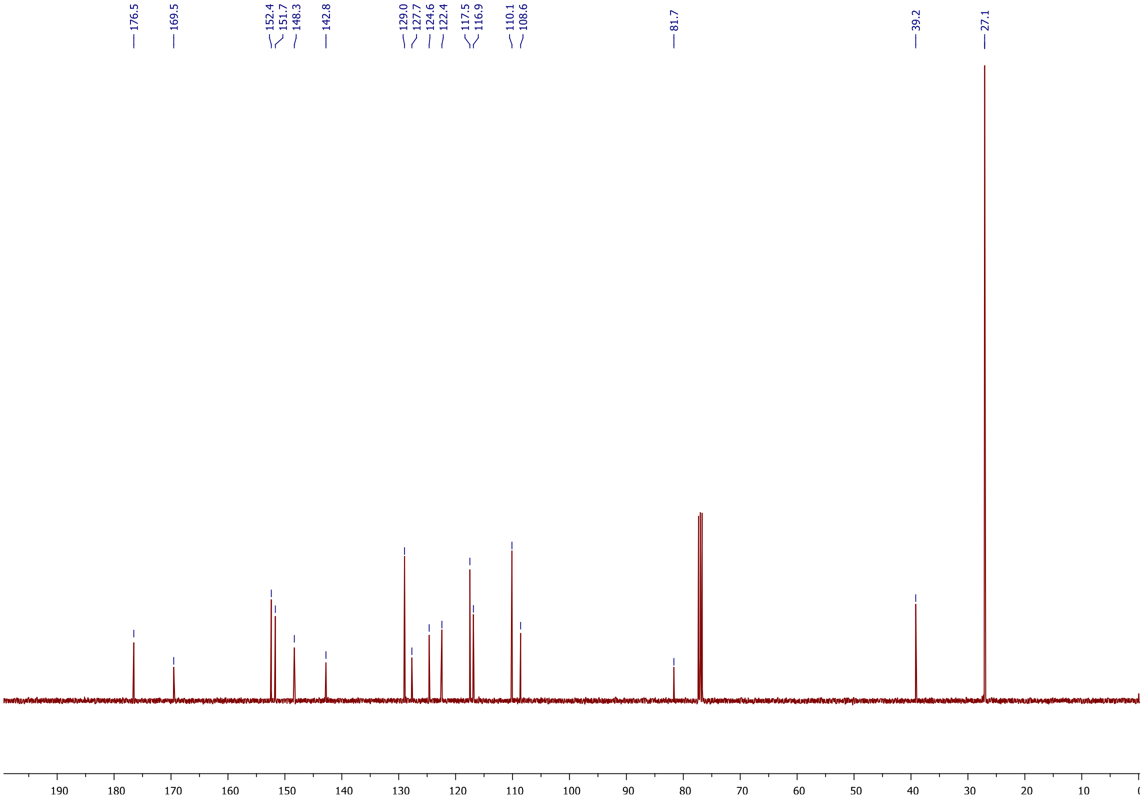

Supplementary Figure S12: ^{1}H and ^{13}C NMR spectra of compound **S2**

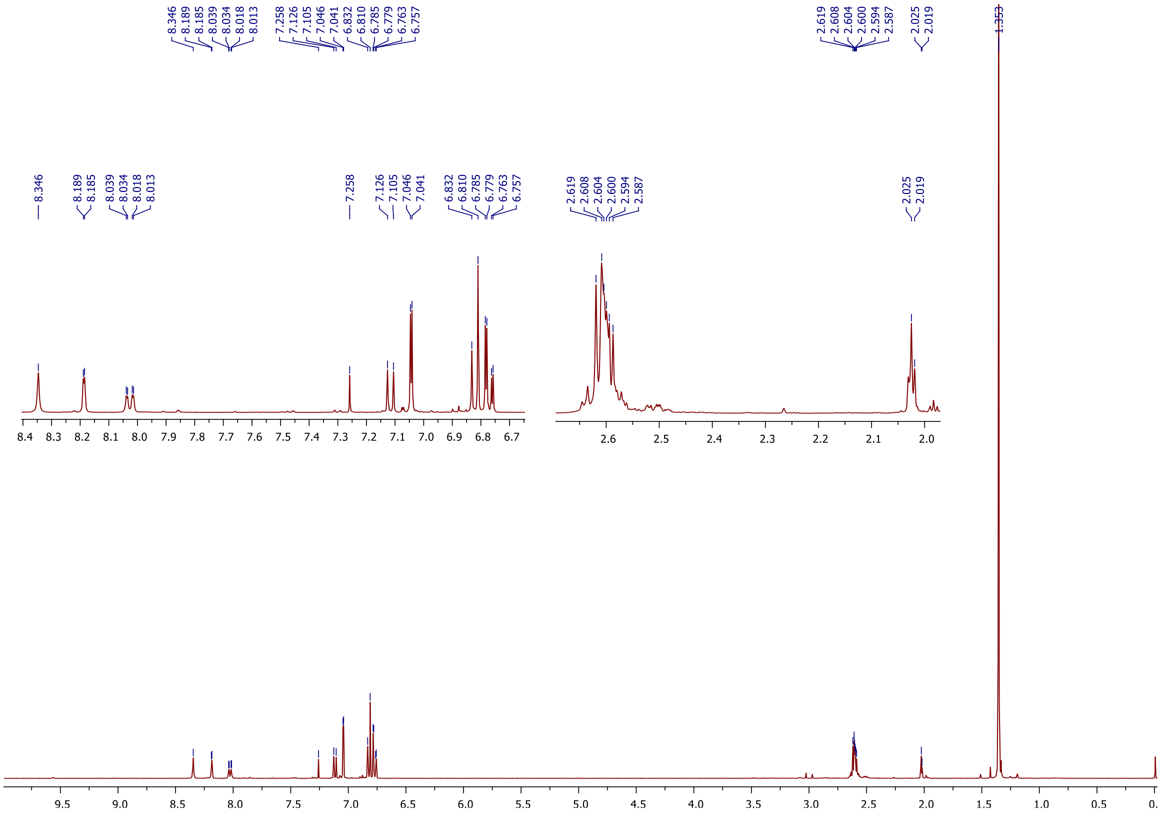

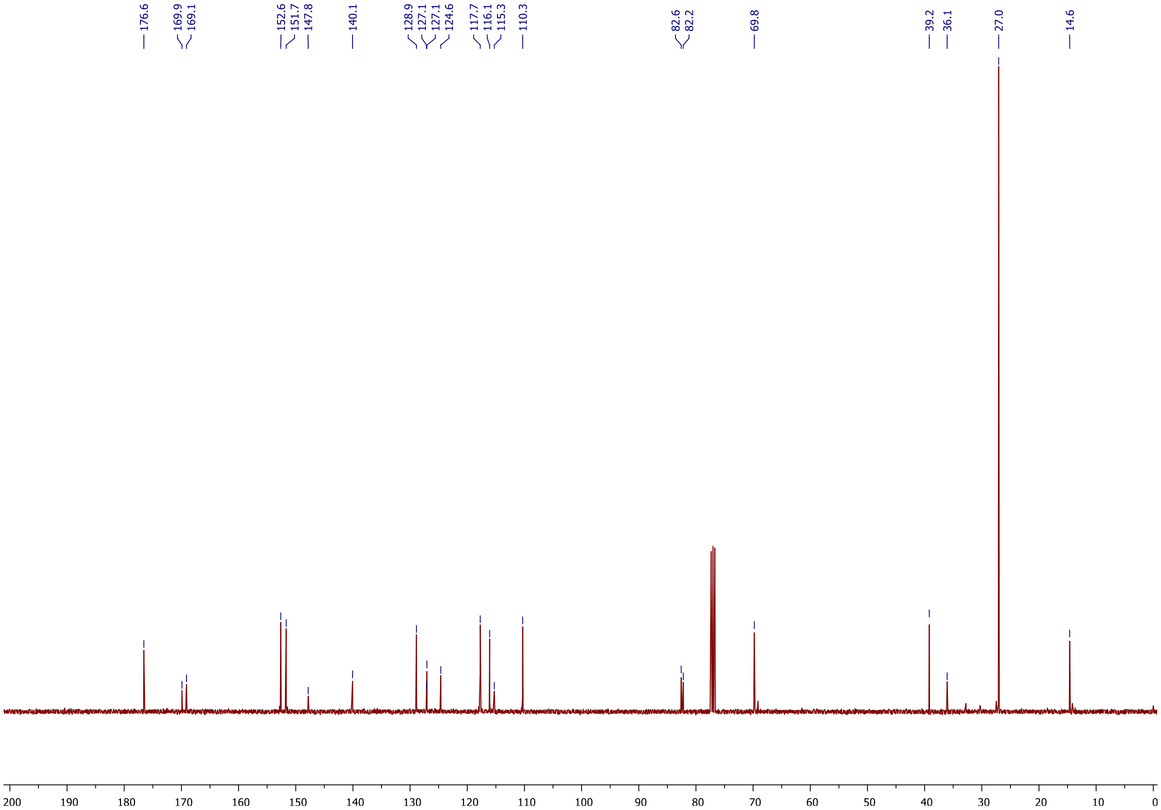

Supplementary Figure S13: ^{1}H and ^{13}C NMR spectra of compound **2**

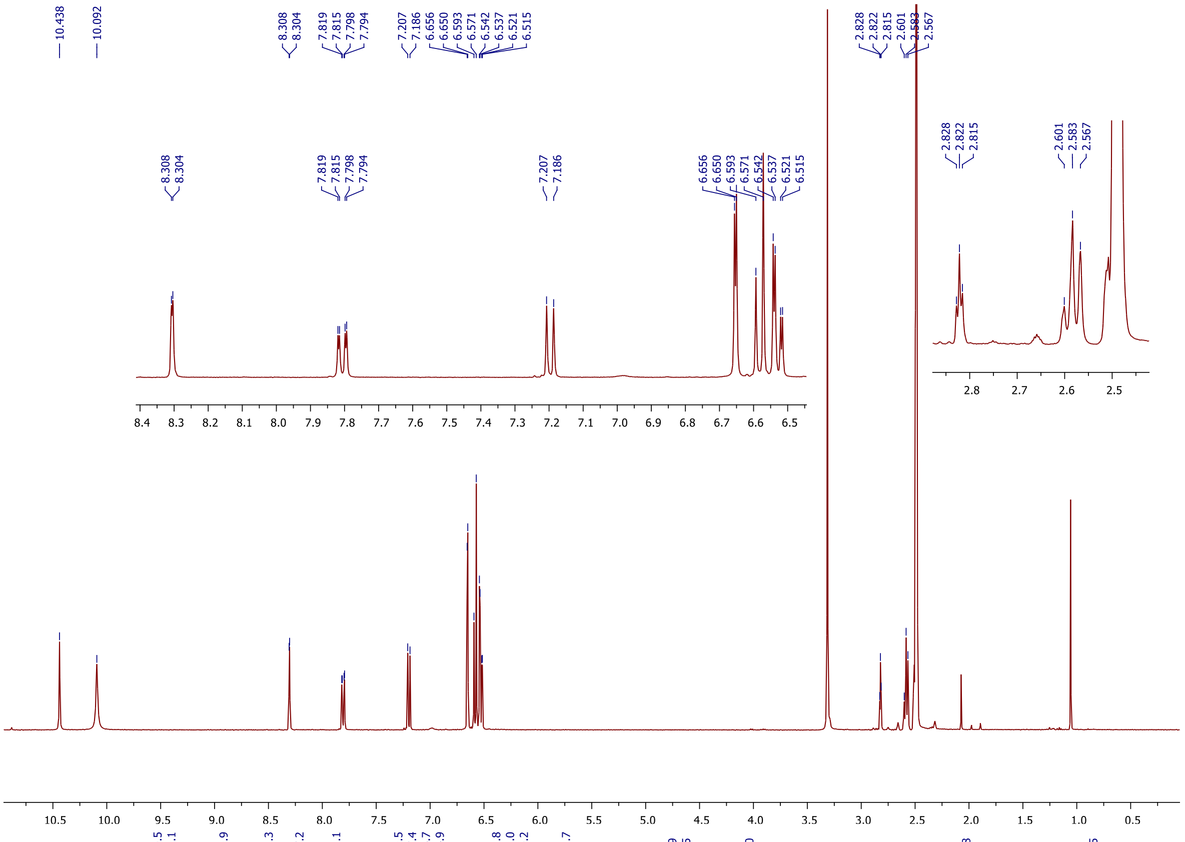

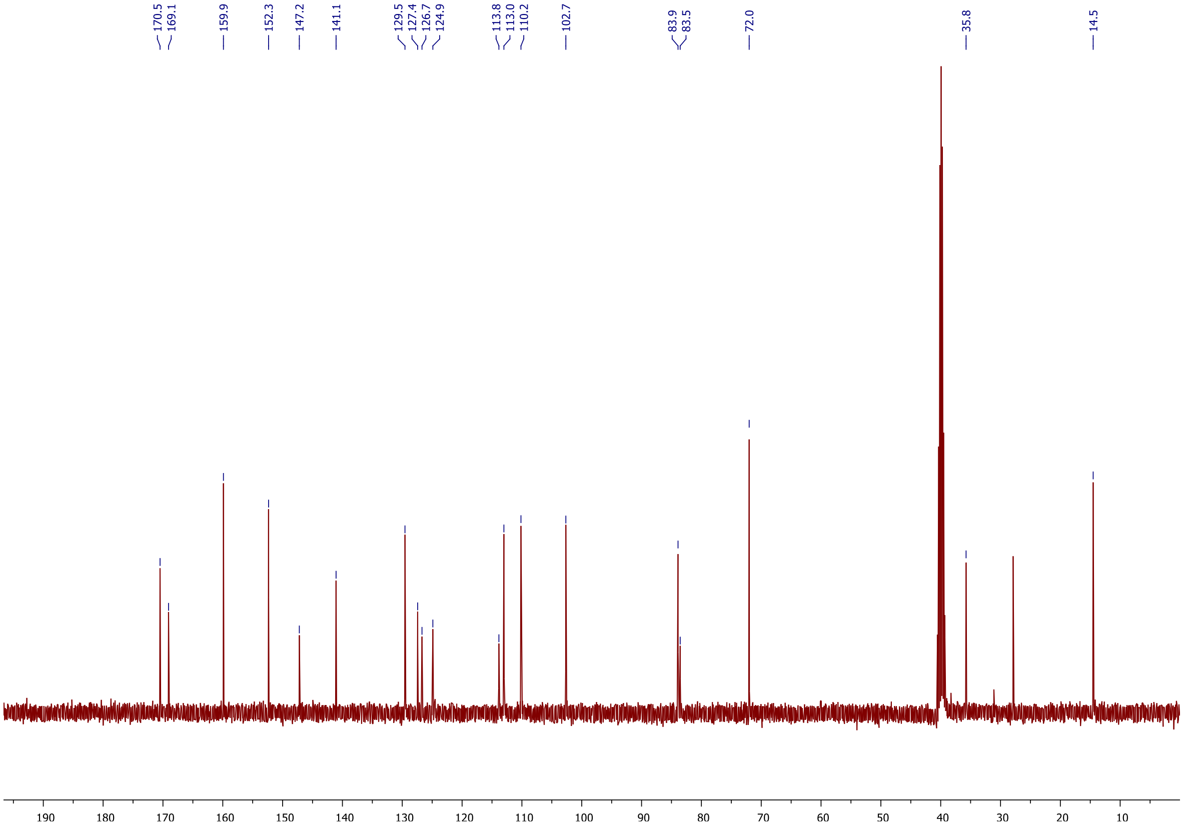

Supplementary Figure S11: ^{1}H, ^{13}C and ^{19}F NMR spectra of **TPF**

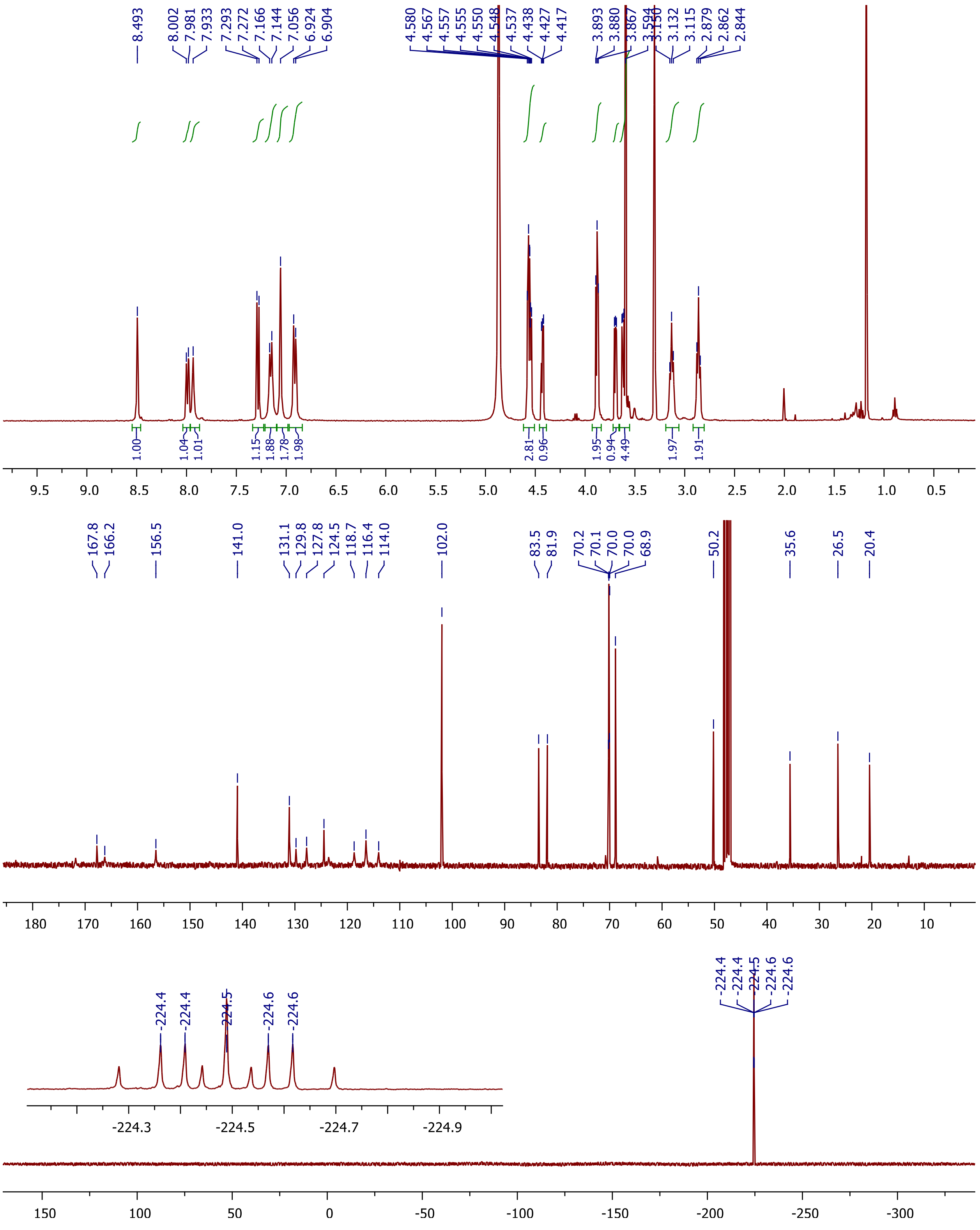

Supplement: Supplementary file 1 — Additional file 1. General methods, Synthetic procedures, Radiolabeling procedures, NMR Spectra of the products, Data on the cell lines, Antibody construction and production. Additional file 1: Figure S1. Binding capacity of bsAb EpCAMxFITC for both EpCAM (A) and fluorescein (B). Additional file 1: Figure S2: bsAb EpCAMxFITC shows dose-dependent capacity to quench the fluorescein-mediated fluorescent activity of TPF, Additional file 1: Figure S3: Capacity of free bsAb EpCAMxFITC and cancer cell-bound bsAb EpCAMxFITC to capture TPF and quench its fluorescence. Additional file 1: Figure S4: Radio-TLC after the fluorination reaction to obtain [18F]3 (eluent: EtOAc/Hexane, 1/1). Additional file 1: Figure S5: HPLC Chromatogram of the purification of [18F]3. UV detector λ = 254 nm. Additional file 1: Figure S6: HPLC Chromatogram of the purification of [18F]TPF. UV detector λ = 312 nm. Additional file 1: Figure S7: HPLC Chromatogram of the collected peak for [18F]TPF, co-injected with the non-radiolabelled reference compound TPF. Additional file 1: Figure S8: Stability of [18F]TPF in human serum. Additional file 1: Figure S9: Assessment of the capacity of bead-bound bsAb EpCAMxFITC to capture [18F]TPF. Additional file 1: Figure S10: 8 wells permanox chamber slide with seeded cells. Additional file 1: Figure S11: 1H and 13C NMR spectra of compound 1. Additional file 1: Figure S12: 1H and 13C NMR spectra of compound S2. Additional file 1: Figure S13: 1H and 13C NMR spectra of compound 2. Additional file 1: Figure S11: 1H, 13C and 19F NMR spectra of TPF. [file 41181_2022_155_MOESM1_ESM.pdf]
